# Sex and Age-Dependent Effects of miR-15a/16-1 Antagomir on Ischemic Stroke Outcomes

**DOI:** 10.3390/ijms252111765

**Published:** 2024-11-01

**Authors:** Xinlei Huang, Shun Li, Na Qiu, Andrew Ni, Tianqing Xiong, Jia Xue, Ke-Jie Yin

**Affiliations:** 1 Department of Neurology, University of Pittsburgh School of Medicine, Pittsburgh, PA 15213, USA; huangx5@upmc.edu (X.H.); lis24@upmc.edu (S.L.); qiun@upmc.edu (N.Q.); andrew_ni@brown.edu (A.N.); xiongt2@upmc.edu (T.X.); xuej5@upmc.edu (J.X.); 2 Geriatric Research, Education and Clinical Center, Veterans Affairs Pittsburgh Healthcare System, Pittsburgh, PA 15240, USA

**Keywords:** antagomir, sex and age, stroke, MCAO, microRNA

## Abstract

Ischemic stroke is a leading cause of disability and mortality worldwide. Recently, increasing evidence implicates microRNAs (miRs) in the pathophysiology of ischemic stroke. Studies have shown that miR-15a/16-1 is abnormally expressed in brains after ischemic stroke, and its upregulation may increase ischemic damage. Given that sex and age are significant modifiers of stroke outcomes, here we investigated whether inhibiting miR-15a/16-1 with antagomirs mitigates cerebral ischemia/reperfusion (I/R) injury in a sex- and age-dependent manner. Young (3 months) and aged (18 months) male and female C57/BL mice underwent 1-h middle cerebral artery occlusion and 3–7 days reperfusion (tMCAO). We administered miR-15a/16-1 antagomir (30 pmol/g) or control antagomir (NC, 30 pmol/g) via tail vein 2 h post-MCAO. Neurobehavioral testing and infarct volume assessment were performed on days 3 and 7. Compared to controls, antagomir treatment significantly improved neurobehavioral outcomes and reduced infarct volume in tMCAO mice at day 7, with the effects being more pronounced in young mice. Notably, young female mice exhibited superior survival and sensorimotor function compared to young male mice. These results were also replicated in a permanent MCAO (pMCAO) mice model. This suggests miR-15a/16-1 antagomir and estradiol may synergistically regulate genes involved in neurovascular cell death, inflammation, and oxidative stress, with sex and age-dependent expression of miR-15a/16-1 and its targets likely underlying the observed variations. Overall, our findings identify miR-15a/16-1 antagomir as a promising therapeutic for ischemic stroke and suggest that sex and age should be considered when developing miR-based therapeutic strategies.

## 1. Introduction

Stroke remains the third leading cause of death and the foremost cause of long-term disability in adults worldwide [1]. It is characterized by a severe reduction in focal cerebral blood flow, leading to neuronal death and infarction in affected brain tissues. While reperfusion therapeutics like tissue plasminogen activator (tPA) and endovascular thrombectomy offer benefits, their effectiveness is limited by strict eligibility criteria and narrow therapeutic windows, leaving many patients without effective treatment options [2,3,4]. Indeed, many patients still suffer severe brain impairment post-ischemic stroke and require functional recovery as a central component for poststroke rehabilitation [5]. Consequently, there is an urgent need for novel therapeutic strategies to mitigate ischemic damage and improve outcomes, particularly for patients with non-reperfusable arteries [6].

MicroRNAs (miRs) are a class of small non-coding RNAs that play crucial regulatory roles in various cellular processes [7]. They are about 21–25 nucleotides in length and exert their effects by targeting specific mRNAs and suppressing their translation [8,9]. Recently, they have emerged as key modulators in central nervous system injuries, including ischemic stroke, with potential roles as both biomarkers and therapeutic targets [10,11,12,13]. Neuroscientists have unraveled molecular mechanisms with microRNA involvement in stroke onset, progression, and outcomes [14,15]. Their findings reveal that miRs exert a profound influence on stroke outcomes by modulating critical signaling pathways that govern cell death, inflammatory responses, oxidative stress, and neuroprotection [16,17]. Among these miRs, miR-15a and miR-16-1 form a highly conserved cluster (miR-15a/16-1 cluster) with a well-established role in various tissues [18]. In the context of ischemic stroke, the miR-15a/16-1 cluster has been implicated in regulating neuroprotection and cerebrovascular function [19,20].

Emerging evidence suggests that the expression and function of microRNA, including miR-15a/16-1, may exhibit sex and age-related variations [21]. This differential regulation could potentially influence the therapeutic efficacy of miR-based interventions such as miR-15a/16-1 antagomir therapy. The regulatory role of sex hormones, such as estrogen and testosterone, in modulating microRNA expression and function is well established. This modulation can contribute to sex differences in microRNA profiles and their associated biological effects. Moreover, the interplay between sex hormones, age, and microRNA function could further influence microRNA regulation and its downstream effects, contributing to the heterogeneity of stroke outcomes observed in different patient populations [22]. However, the sex- and age-dependent effects of miR-15a/16-1 inhibition in ischemic stroke remain poorly understood.

## 2. Results

### 2.1. Experimental Flowchart

**Figure 1:**
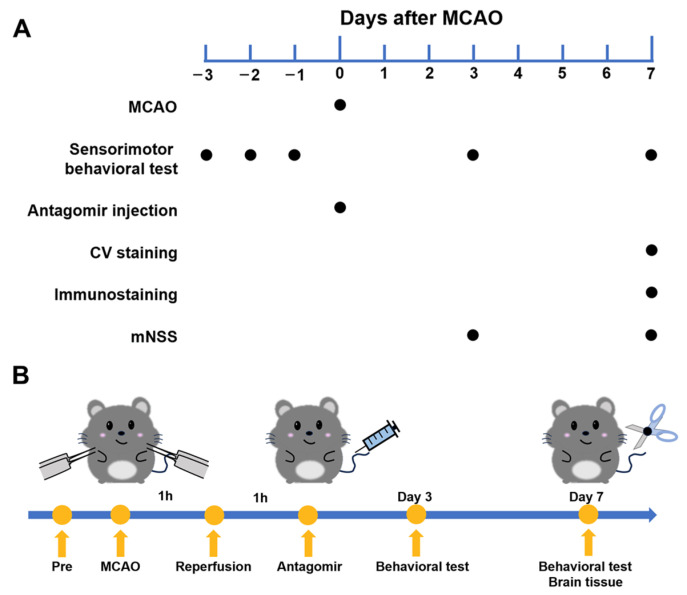
Schematic representation of the experimental procedure. (**A**) The timeline of key experimental events, including MCAO, antagomir injection, sensorimotor behavioral tests, and subsequent analyses over a 7-day period post-MCAO. (**B**) Schematic diagram of the detailed sequence of the experimental program.

We designed experiments and devised flow charts regarding MCAO surgery, behavioral tests, antagomir injections, and histological analyses (Figure 1A,B) to study the effects of stroke.

### 2.2. miR-15a/16-1 Antagomir Improves Sensorimotor Behavior in Mice After tMCAO

**Figure 2:**
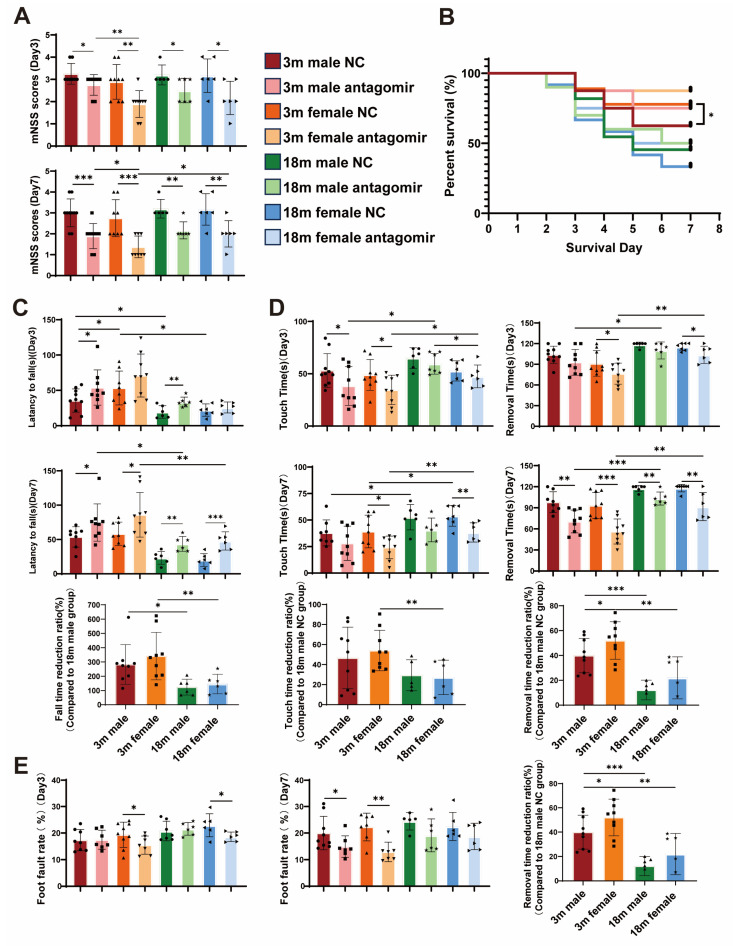
miR-15a/16-1 antagomir improves sensorimotor behavior in mice after tMCAO. We analyzed the effects of miR-15a/16-1 antagomir treatment on neurobehavioral outcomes, survival, and sensorimotor functions in young (3-month-old) and aged (18-month-old) male and female mice following tMCAO. (**A**) mNSS was evaluated on Day 3 and Day 7 post-tMCAO; miR-15a/16-1 antagomir-treated groups showed significant improvements compared to controls (n = 6–10). (**B**) Survival analysis indicates improved survival rates in antagomir-treated mice, particularly in younger females (n = 9–12). (**C**–**E**) Sensorimotor function was assessed using various tests, including rotarod, foot-fault and adhesive tape removal, revealing that miR-15a/16-1 antagomir treatment significantly enhanced performance in these tasks, particularly in young females, on both Day 3 and Day 7 post-tMCAO (n = 6–9). Data were analyzed using one-way ANOVA followed by *post hoc* tests. Significant differences were noted among the groups (*** *p* < 0.001, ** *p* < 0.01, * *p* < 0.05).

We investigated the effects of miR-15a/16-1 antagomir on functional recovery following transient middle cerebral artery occlusion (tMCAO) in mice. In our study, a 2 × 2 × 2 factorial design was used to examine the effects of three factors—sex (male, female), age (young, aged) and treatment (miR-15a/16-1 NC, miR-15a/16-1 antagomir) on the experimental results. The treatment was administered through the tail vein 2 h post-stroke. Survival rate and neurological deficit were monitored, and sensorimotor behavior was assessed (Figure 2A,B). Statistical analysis indicated that the modified neurological severity score (mNSS) of mice was significantly reduced after injection of miR-15a/16-1 antagomir, and there was a significant difference in the survival rate of mice in the young female treatment group compared to the NC group. Furthermore, sensorimotor behavior tests showed marked improvement in each treatment group compared to each control (Figure 2C–E). Mice treated with the antagomir demonstrated better coordination and balance, indicating enhanced functional recovery. Additionally, the survival rate in the aged NC group was lower compared to the young NC group, with higher mNSS and poorer sensorimotor behavior. Among the young mice, females exhibited significantly better outcomes post-treatment, suggesting a sex-specific enhancement in neuroprotection and functional recovery.

### 2.3. miR-15a/16-1 Antagomir Reduces Brain Infarct Volume in Mice 7 Days After tMCAO

**Figure 3:**
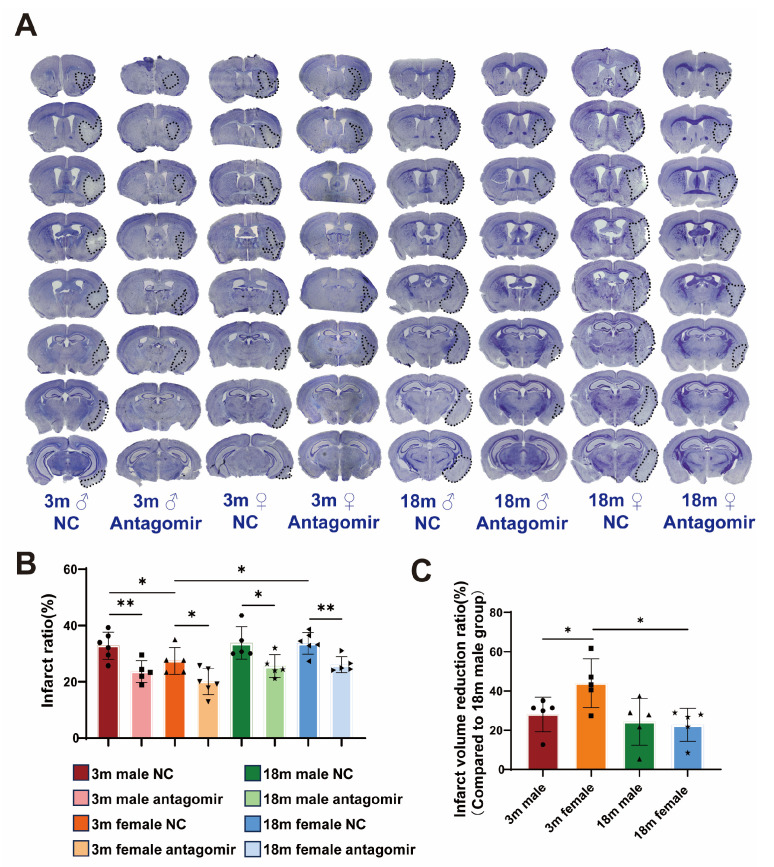
miR-15a/16-1 antagomir reduces brain infarct volume in mice 7 days after tMCAO. (**A**) Representative images of CV staining, showing the infarct volume (outlined in black) in both NC and miR-15a/16-1 antagomir-treated groups across different ages (3 months and 18 months) and sexes (male and female) at 7 days post-tMCAO. (**B**) Quantitative analysis of infarct ratio (%) shows a significant reduction in infarct volume in antagomir-treated groups compared to controls, with more pronounced effects in younger and female mice (n = 5–6). (**C**) Infarct volume reduction ratio (%) is presented, demonstrating significant differences in response to treatment among different groups (n = 5). Data were analyzed using one-way ANOVA followed by *post hoc* tests, with statistical significance indicated (* *p* < 0.05, ** *p* < 0.01).

To evaluate the neuroprotective effects of miR-15a/16-1 antagomir treatment on ischemic stroke outcomes, we performed CV staining (Figure 3A) to assess damage in ischemic brain regions. Histological analysis (Figure 3B,C) reveals that NC groups exhibit significant infarct volume across all sex and age categories, while mice treated with miR-15a/16-1 antagomir display markedly reduced infarct volume, indicating the neuroprotective effect of the treatment. Quantitative analysis shows that young male and young female mice have significantly lower infarct ratios following antagomir treatment, with young females showing the most substantial reduction in infarct volume. In contrast, aged male and female mice also exhibit reduced infarct ratios, but the effects are less pronounced. Overall, miR-15a/16-1 antagomir treatment significantly decreases infarct volume in different age and sex groups, with the most notable improvements observed in young mice, particularly females.

### 2.4. miR-15a/16-1 Antagomir Reduces Neuronal Loss Volume in Mice 7 Days After tMCAO

**Figure 4:**
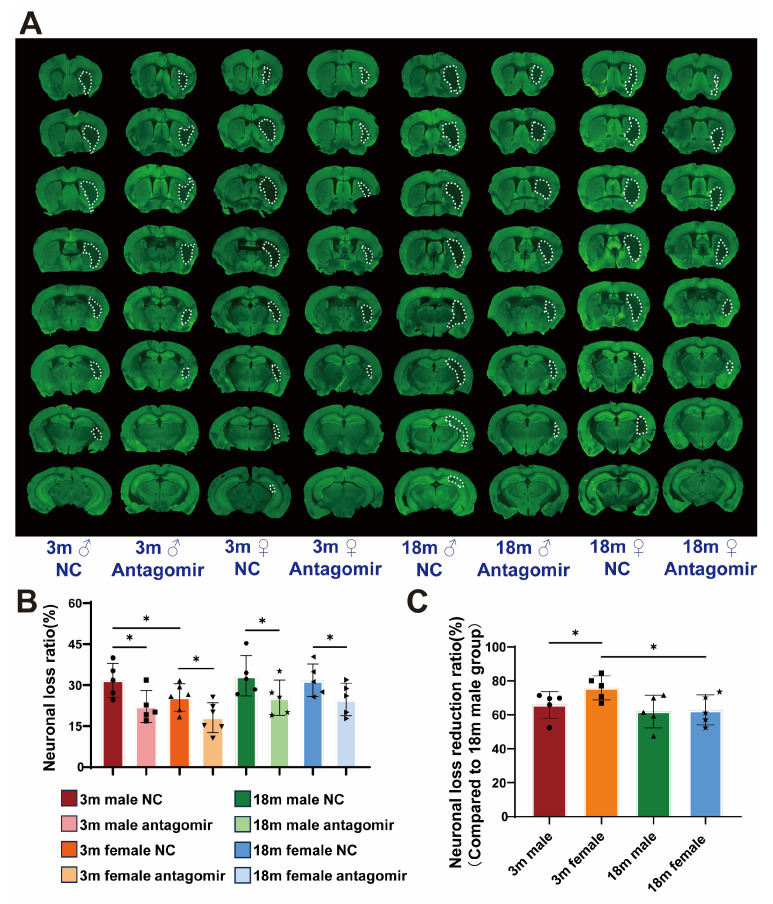
miR-15a/16-1 antagomir reduces neuronal loss volume in mice 7 days after tMCAO. (**A**) Representative images of MAP2 immunostaining, showing the volume of neuronal loss (outlined in white) in different groups at 7 days post-tMCAO. (**B**) Quantitative analysis of neuronal loss ratio (%) reveals a significant reduction in neuronal loss in antagomir-treated groups compared to controls, with greater efficacy observed in younger and female mice (n = 5–6). (**C**) Neuronal loss reduction ratio (%) is presented, demonstrating significant differences in response to treatment among different groups, with a clear influence of age and sex on the therapeutic outcomes (n = 5). Data were analyzed using one-way ANOVA followed by *post hoc* tests, with statistical significance indicated (* *p* < 0.05).

Since, neuronal loss volume is the most straightforward indicator for assessing the severity of cerebral ischemia, we performed MAP2 immunostaining (Figure 4A). The results (Figure 4B,C), consistent with our previous findings, show that NC groups exhibit significant neuronal loss across all age and sex categories. In contrast, mice treated with miR-15a/16-1 antagomir display markedly reduced neuronal loss volumes. Young male and young female mice show significantly lower neuronal loss following antagomir treatment, with young females demonstrating the most substantial reduction. Aged male and female mice also exhibit reduced neuronal loss, but the effect is less pronounced compared to younger mice. These results indicate that miR-15a/16-1 antagomir treatment effectively reduces neuronal loss volume in ischemic stroke, particularly in young mice, with females showing the greatest improvement.

### 2.5. miR-15a/16-1 Antagomir Improves Sensorimotor Behavior in Mice After pMCAO

**Figure 5:**
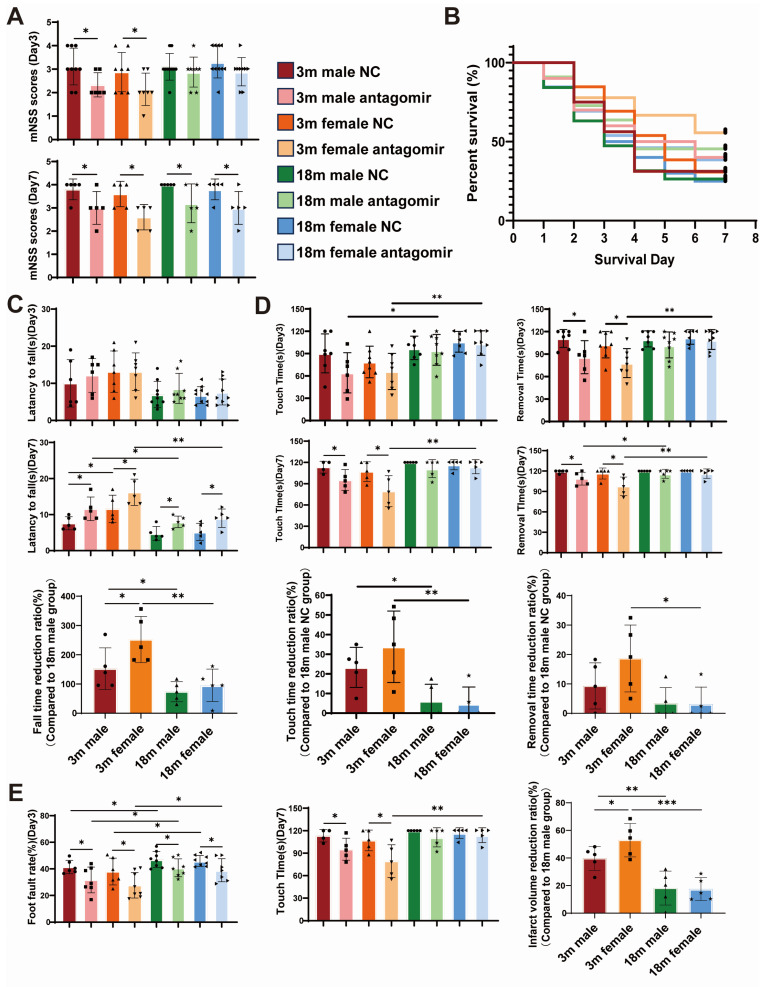
miR-15a/16-1 antagomir improves sensorimotor behavior in mice after pMCAO. (**A**) mNSS was assessed on Day 3 and Day 7 post-pMCAO, showing significant improvements in the antagomir-treated groups compared to NC groups, with differences observed based on age and sex (n = 5–10). (**B**) Survival curves indicate varied survival rates among the different groups (n = 10–20). (**C**–**E**) Sensorimotor functions were evaluated through rotarod, foot-fault and adhesive tape removal tests, revealing that miR-15a/16-1 antagomir treatment significantly enhanced performance, particularly in younger female mice, across all tested parameters (n = 5–10). Data were analyzed using one-way ANOVA followed by *post hoc* tests, with significant differences denoted (* *p* < 0.05, ** *p* < 0.01, *** *p* < 0.001).

Next, we extended our investigation to the permanent MCAO (pMCAO) model with the same factorial design. Statistical analysis (Figure 5A) revealed that the mNSS scores of mice were significantly reduced following the injection of miR-15a/16-1 antagomir. However, we did not find a significant difference in survival rates (Figure 5B) in the pMCAO model. Among the young mice, females exhibited significantly better outcomes post-treatment, suggesting a sex-specific enhancement in neuroprotection and functional recovery (Figure 5C–E). In summary, miR-15a/16-1 antagomir treatment effectively improves sensorimotor behavior in pMCAO models, with the most pronounced benefits observed in young female mice, highlighting the importance of considering both age and sex in therapeutic interventions. These findings underscore the efficacy of miR-15a/16-1 antagomir in reducing neurological deficits in two stroke models, with the best efficacy in young female mice in both models.

### 2.6. miR-15a/16-1 Antagomir Reduces Brain Infarct Volume in Mice 7 Days After pMCAO

**Figure 6:**
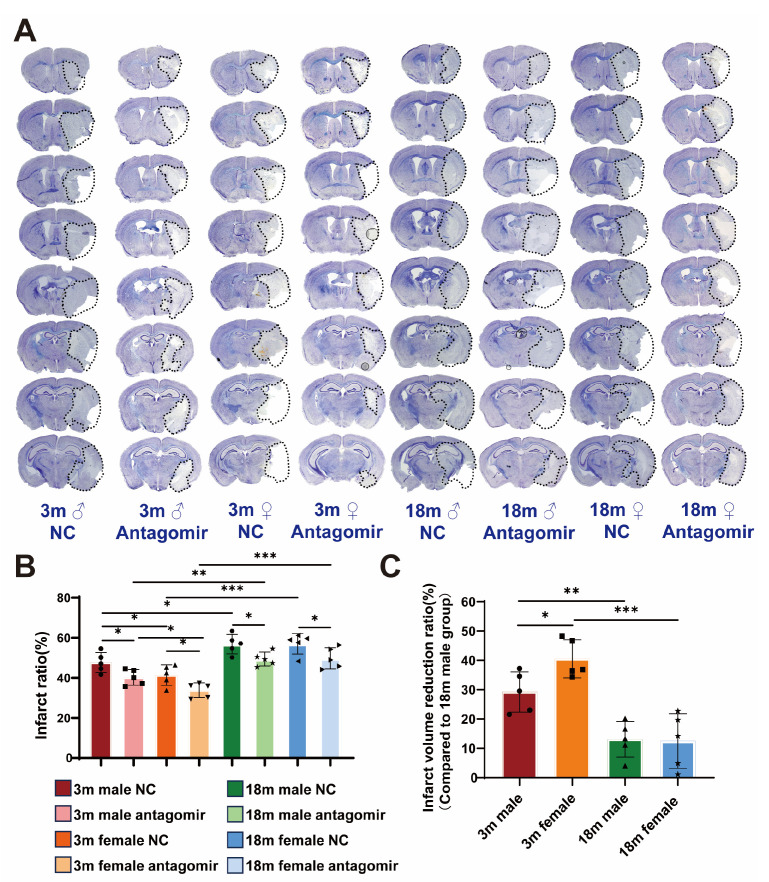
miR-15a/16-1 antagomir reduces brain infarct volume in mice 7 days after pMCAO. (**A**) Representative images of CV staining, highlighting the infarct volume (outlined in black) in different groups at 7 days post-pMCAO. (**B**) Quantitative analysis of infarct ratio (%) reveals a significant reduction in infarct volume in antagomir-treated groups compared to NC groups, with more pronounced effects observed in younger and female mice (n = 5). (**C**) Infarct volume reduction ratio (%) is presented, showing significant differences in treatment response across different groups (n = 5). Statistical analysis was performed using one-way ANOVA followed by *post hoc* tests, with significance levels indicated (*** *p* < 0.001, ** *p* < 0.01, * *p* < 0.05).

The results of CV staining (Figure 6A) demonstrate that miR-15a/16-1 antagomir significantly reduces brain infarct volume in mice 7 days after pMCAO. As shown in Figure 6B,C, the infarct ratio (%) is substantially lower in the treatment groups compared to the NC groups across young and aged, male and female mice. Notably, young female mice treated with the antagomir exhibit the greatest reduction in infarct volume, followed by young male mice. Differences in therapeutic efficacy between aged mice of different sexes do not appear to be significant. Further analysis reveals a significant reduction in infarct volume, with young females showing approximately three times the reduction compared to the reference group of aged males. These findings indicate that miR-15a/16-1 antagomir treatment effectively mitigates ischemic brain injury, with the most pronounced neuroprotection observed in young female mice.

### 2.7. miR-15a/16-1 Antagomir Reduces Neuronal Loss Volume in Mouse 7 Days After pMCAO

**Figure 7:**
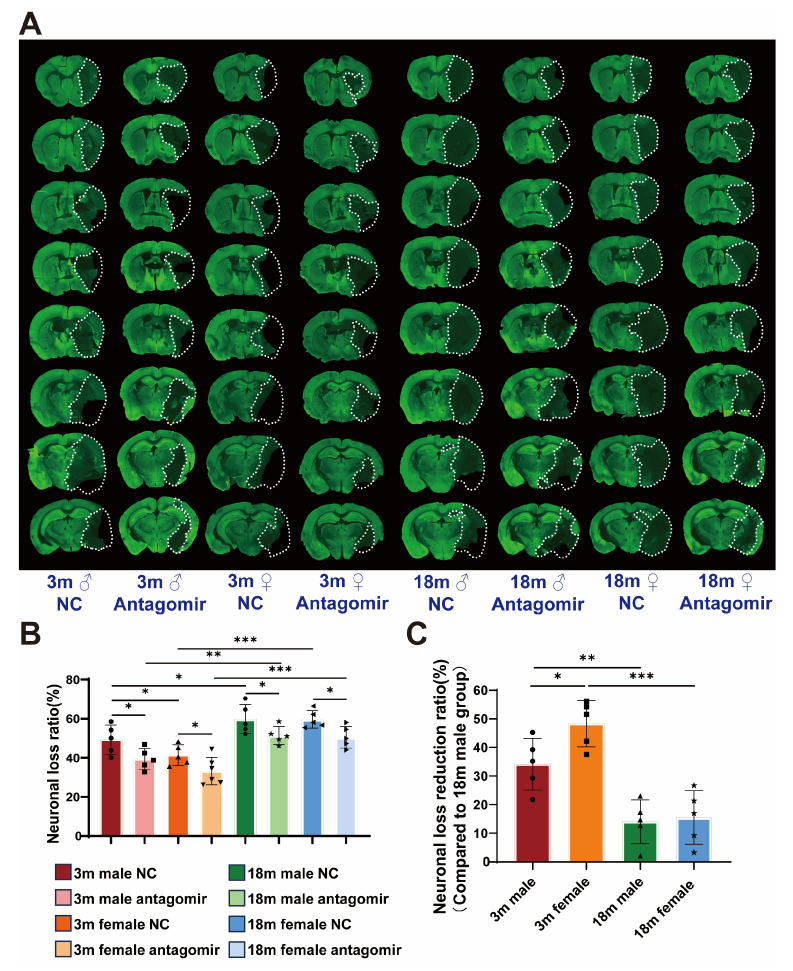
miR-15a/16-1 antagomir reduces neuronal loss volume in mice 7 days after pMCAO. (**A**) Representative images of MAP2 immunostaining, showing the volume of neuronal loss (outlined in white) in different groups at 7 days post-pMCAO. (**B**) Quantitative analysis of neuronal loss ratio (%) demonstrates a significant reduction in neuronal loss in antagomir-treated groups compared to controls, with more pronounced effects observed in younger and female mice (n = 5). (**C**) Neuronal loss reduction ratio (%) is presented, showing significant differences in treatment response across different groups (n = 5). Statistical analysis was performed using one-way ANOVA followed by *post hoc* tests, with significance levels indicated (*** *p* < 0.001, ** *p* < 0.01, * *p* < 0.05).

To further investigate the impact of miR-15a/16-1 antagomir on neuronal survival following pMCAO, we utilized MAP2 immunostaining (Figure 7A). The results indicated significant differences in neuronal loss volume between the NC and treatment groups (Figure 7B). Notably, the young female group exhibited smaller neuronal loss volume compared to the young male group, while no significant differences were observed between the aged female and male groups. Additionally, both sexes in the young groups had smaller neuronal loss volumes compared to their aged counterparts. All treatment groups showed a significant reduction in neuronal loss volume compared to their respective NC groups. Using the aged male group as a reference, the young female group demonstrated the most effective treatment outcome, approximately three times that of the aged male group. Followed by the young male group, with about twice the effect, both with significant differences (Figure 7C). The aged groups, regardless of sex, exhibited poorer treatment efficacy, with no significant differences between sexes. The results suggest that miR-15a/16-1 antagomir treatment substantially enhances neuronal survival post-pMCAO, particularly in young mice.

## 3. Discussion

Our previous research confirmed that inhibiting miR-15a/16-1 improves prognosis in ischemic stroke [19,23,24]. Building on these findings, the current study investigates the therapeutic effects of miR-15a/16-1 antagomir across two different stroke models, focusing on its ability to enhance neurobehavioral outcomes, reduce infarct volume, and decrease neuronal death in mice of varying ages and sexes [15]. Notably, young female mice exhibited the most significant improvements, indicating a sex- and age-dependent therapeutic efficacy of miR-15a/16-1 inhibition. This study aims to explore the differential efficacy of miR-15a/16-1 antagomir treatment, providing insights into how age and sex influence the therapeutic response in ischemic stroke [22].

Estradiol, a form of estrogen, is well-documented for its neuroprotective effects, particularly in females. Research indicates that higher levels of estradiol are associated with improved functional recovery following ischemic stroke, likely due to its anti-apoptotic, anti-inflammatory, and antioxidative properties [25,26]. As estradiol levels decline with age—especially during menopause—the protective effects diminish, which may explain the reduced efficacy observed in aged female mice [27]. Conversely, while testosterone is generally linked with positive outcomes in males, its role in females is more complex and less understood. High testosterone levels in males are associated with better post-stroke outcomes, yet the age-related decline in testosterone also influences stroke prognosis. Nonetheless, the diminished hormone levels in aged mice, regardless of sex, could contribute to the attenuated response to treatment. Overall, understanding the interplay between sex hormones and stroke pathology is crucial for developing effective therapeutic strategies that account for sex and age differences.

In our experiments, we presented substantial evidence demonstrating that young female mice respond most favorably to miR-15a/16-1 antagomir treatment, indicating potential sex-specific mechanisms of neuroprotection [28,29]. Conversely, the diminished efficacy observed in aged mice highlights the need for tailored approaches to optimize treatment outcomes across different age groups. It is also worth noting that in the younger groups, female mice exhibited better outcomes both before and after treatment compared to their male counterparts, whereas no significant differences were observed in the aged groups before and after treatment. This suggests that estradiol (estrogen) may provide greater neuroprotection in ischemic stroke than testosterone (androgen), and the decline of both hormones’ levels with age may exacerbate stroke severity [30]. Despite these age-related declines, miR-15a/16-1 antagomir treatment was still effective in improving outcomes, highlighting its potential as a therapeutic intervention across different age and sex groups. This body of research underscores the importance of considering both sex and age in the development of effective stroke therapies.

In summary, our study showed that miR-15a/16-1 antagomir treatment significantly enhances neurobehavioral recovery, reduces infarct volume, and mitigates neuronal death in ischemic stroke, with the most pronounced benefits observed in young female mice. These findings suggest that the neuroprotective effects of estradiol, which are more prominent in younger females, may be a critical factor in the differential response to treatment [28,29,30]. Furthermore, the observed diminished efficacy in aged mice, regardless of sex, highlights the need for age-specific strategies or adjunct therapies to enhance treatment benefits in older populations. Our research emphasizes the need to account for sex and age when designing stroke interventions, as these factors significantly influence the therapeutic outcomes of miR-15a/16-1 inhibition. The promising results of miR-15a/16-1 antagomir across diverse demographic groups highlight its potential as a versatile and effective treatment option for ischemic stroke. Future research should focus on elucidating the underlying mechanisms that drive these sex and age-dependent differences to further optimize therapeutic approaches.

## 4. Materials and Methods

### 4.1. Animals

Young C57BL/6J mice (females and males, 3 months, 23–28 g) were purchased from The Jackson Laboratory (Bar Harbor, ME, USA). Aged C57BL/6J mice (females and males, 18 months, 28–38 g) were provided by National Institute on Aging (NIA, Wilmington, MA, USA). All procedures were approved by the University of Pittsburgh Institutional Animal Care and Use Committee and performed in accordance with the National Institutes of Health Guide for the Care and Use of Laboratory Animals. All experiments were reported in compliance with the ARRIVE guidelines 2.0 (ARRIVE, Animal Research: Reporting in Vivo Experiments) [31]. All mice were housed in a room where the lighting was controlled (12 h on, 12 h off) and the room temperature was kept around 22 °C. Mice were given a standard diet and water ad libitum. All efforts were made to minimize animal suffering and the number of experimental animals used.

### 4.2. Mouse Model of Transient/Permanent Focal Cerebral Ischemia

Focal cerebral ischemia was induced by transient/permanent middle cerebral artery occlusion (tMCAO/pMCAO). Briefly, young (3 months, 23–28 g) and aged (18 months, 28–38 g) mice were anesthetized with isoflurane (3% for induction, 1.5% for maintenance) in mixed O_2_ and N_2_O (30%:67%). After a midline skin incision, the common carotid artery was exposed, and its branches were electro-coagulated. A 1.3-cm length of a 7-0 rounded-tip nylon suture (Doccol Corporation, Sharon, MA, USA) was gently advanced from the external carotid artery to the internal carotid artery and further to the origin of the middle cerebral artery (MCA) until regional cerebral blood flow (CBF) was reduced to ~25% of baseline. For the tMCAO model, we removed the suture after 60 min of MCA occlusion, and blood flow was restored. After reperfusion from MCAO, the mice were allowed to recover, and body temperature was measured with a rectal thermometer and maintained at 37.0 ± 0.5 °C by a temperature-controlled heating pad during the ischemic period. Regional CBF was measured using a laser speckle imager (Perimed PeriCam PSI HR, Stockholm, Sweden) 15 min before MCAO surgery, 15 min during the MCAO period, and 15 min after the onset of reperfusion. For the pMCAO model, we kept the suture inside until the designated time point. The analgesic ketoprofen (3 mg/kg) was injected intramuscularly into the animals immediately prior to MCAO surgery and twice a day for the following 2 days post-surgery. Animals that fulfilled the exclusion criteria during/after MCAO were excluded from the data analyses.

### 4.3. Intravenous Administration of miR-15a/16-1 Antagomir

After 2 h reperfusion after 1 h MCAO, mice were injected with miR-15a/16-1 antagomir (a mixture of mmu-miR-15a-5p and mmu-miR-16-1 5p, IDT, Coralville, IA, USA, both of them were administered at 30 pmol/g) or control antagomir (NC-5 control, IDT, Coralville, IA, USA, 30 pmol/g) by tail vein. All mice were sacrificed 7 days after MCAO.

### 4.4. Neurobehavioral Tests

The rotarod, foot-fault, and adhesive tape removal tests were performed as we described previously [32] to assess neurological functions 3 and 7 days after MCAO. The mice that died before the endpoints were excluded from the final behavioral analyses.

### 4.5. Cresyl Violet (CV) Staining

CV histological staining was performed to examine MCAO-induced neuronal death [32]. Briefly, brain sections were deionized and immersed in Cresyl Violet solution, then dehydrated in a graded series of distilled water, 70% alcohol, 95% alcohol, 100% alcohol, and xylene, and mounted with a permanent mounting medium. Eight levels of coronal brain sections selected from front to back in the brain were used for examining the volume of CV-stained neurons in each brain. Images were taken with an EVOS M7000 microscope (Thermo Fisher Scientific, Waltham, MA, USA), and the percentage of CV-positive stained volume was calculated and analyzed using ImageJ Software version 1.54k.

### 4.6. MAP2 Immunostaining

MAP2 immunostaining was performed as previously described [32]. Briefly, brain sections were washed 3 times for 5 min with PBS in a 24-well plate, permeabilized once for 20 min with 1% PBST (1% Triton X-100 in PBS), and washed two times for 5 min with 0.3% PBST. The free-floating sections were then blocked with 5% normal donkey serum in 0.3% PBST for 1 h at room temperature. Then, mouse brain sections were incubated with primary antibody (1:500 diluted in 0.3% PBST) overnight at 4 °C, followed by Alexa Fluor VR 488 streptavidin (1:1000, Jackson ImmunoResearch Laboratories, West Grove, PA, USA) for 1 h at room temperature. The sections were washed with 0.3% PBST three times and mounted with an antifade mounting medium. MAP2 immunostaining images of 8 coronal brain sections (bregma: +1.10 mm to −3.28 mm) were visualized using an EVOS M7000 microscope and used for the assessment of MCAO-induced brain atrophy. Brain atrophy (%) = 100 × (contralateral hemisphere volume − ipsilateral hemisphere volume)/contralateral hemisphere volume [20].

### 4.7. Statistics

All data in this study are expressed as mean ± SD with dots and analyzed with GraphPad Prism (Version 9.0.0, San Diego, CA, USA). For data that meet with the Shapiro–Wilk normality test and Brown–Forsythe homogeneity of variance test of multiple-group comparisons, 1-way or 2-way ANOVA was used followed by Tukey’s post hoc test. Survival analysis was conducted using the log-rank test. For comparisons between the two groups, we used A 2-tailed *t*-test. A *p*-value of <0.05 was considered statistically significant.

## 5. Conclusions

Our findings identify miR-15a/16-1 antagomir as a promising therapeutic candidate for alleviating ischemic stroke-induced damage, particularly in younger mice. The differential effects observed between sexes, with young female mice showing superior outcomes compared to their male counterparts, highlight the importance of considering sex-specific responses in therapeutic development. Furthermore, the data show that age highly influences treatment efficacy, suggesting that younger individuals may derive more substantial benefits from miR-15a/16-1 antagomir therapy. These findings provide a foundation for future research aimed at optimizing miR-based therapies for ischemic stroke, with a particular focus on tailoring interventions based on patient sex and age. Future investigations should focus on elucidating the molecular mechanisms underlying these differential responses and exploring the translational potential of miR-15a/16-1 inhibition in clinical settings.

## Data Availability

All data are included in the article. Further information or data can be made available upon request.
